# Species conservation profile of the alpine stenoendemic spider *Vesubia jugorum* (Araneae, Lycosidae) from the Maritime Alps

**DOI:** 10.3897/BDJ.4.e10527

**Published:** 2016-10-07

**Authors:** Stefano Mammola, Filippo Milano, Pedro Cardoso, Marco Isaia

**Affiliations:** ‡ Department of Life Sciences and Systems Biology, University of Torino, Torino, Italy; § IUCN SSC Spider & Scorpion Specialist Group, Torino, Italy; | IUCN SSC Spider & Scorpion Specialist Group, Helsinki, Finland; ¶ Finnish Museum of Natural History, University of Helsinki, Helsinki, Finland

**Keywords:** Climate Change, wolf spider, high altitudes, IUCN, red list

## Species information

Scientific name: Vesubia jugorum

Species authority: (Simon, 1881)

Common names: Giant alpine spider (English)

Kingdom: Animalia

Phylum: Arthropoda

Class: Arachnida

Order: Araneae

Family: Lycosidae

**Figure 1.**
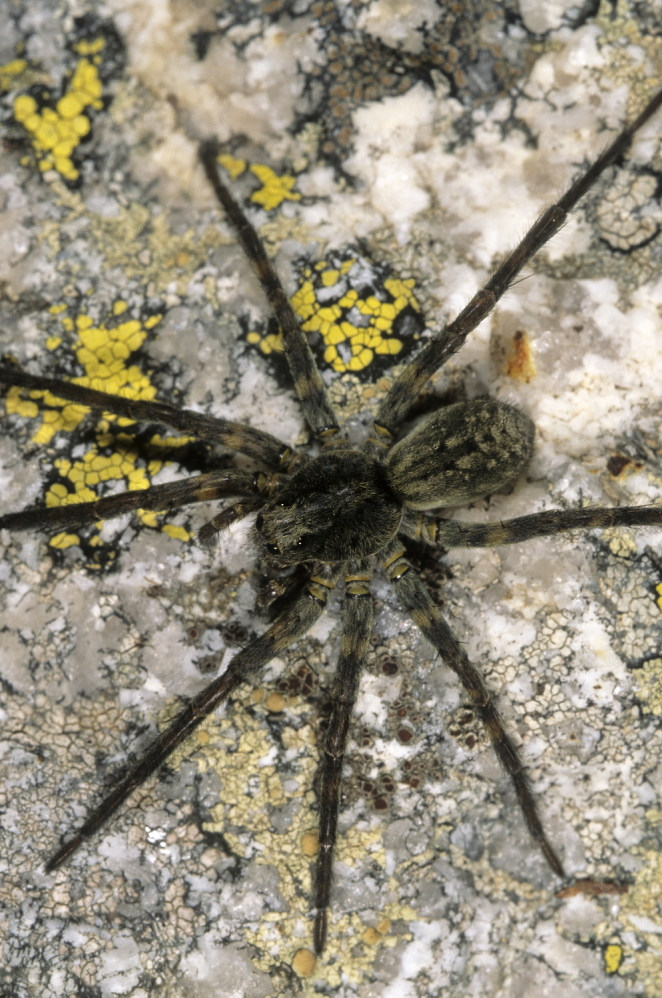
Habitus of *Vesubia jugorum* — female. [Photo credit: Emanuele Biggi, 2003]

**Figure 2.**
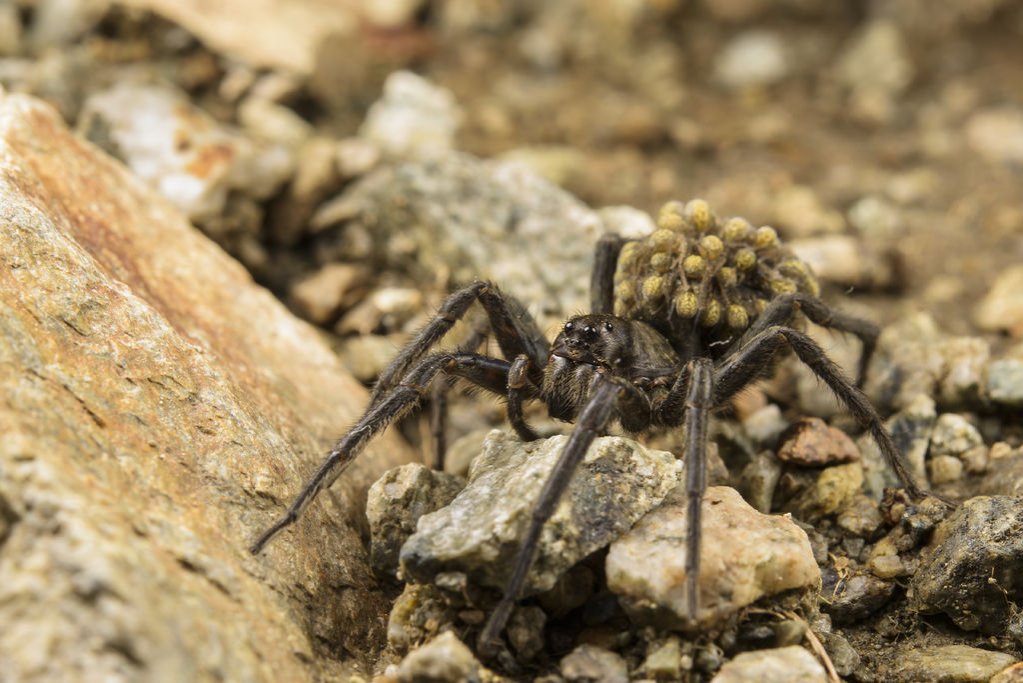
*Vesubia jugorum* — female with spiderlings. [Photo credit: Emanuele Biggi, 2016]

Taxonomic notes: Vesubia jugorum is a large-sized spider (body length: 15–20 mm, prosoma: 7-9 mm). The prosoma is generally blackish or dark brown, marked with black streaks irradiating from the fovea. The opisthosoma is dark grey dorsally and brown reddish ventrally. Legs are dark brown dorsally and reddish-yellowish ventrally, especially on coxae (Figs 1, 2​). See Tongiorgi (1968), Tongiorgi (1969), Maurer and Thaler (1988) and Nentwig et al. (2016) for genitalic drawings and other relevant diagnostic features.

Figure(s) or Photo(s): Figs 2, 4

Region for assessment: Global

## Geographic range

Biogeographic realm: Palearctic

Countries: France

**Figure 3.**
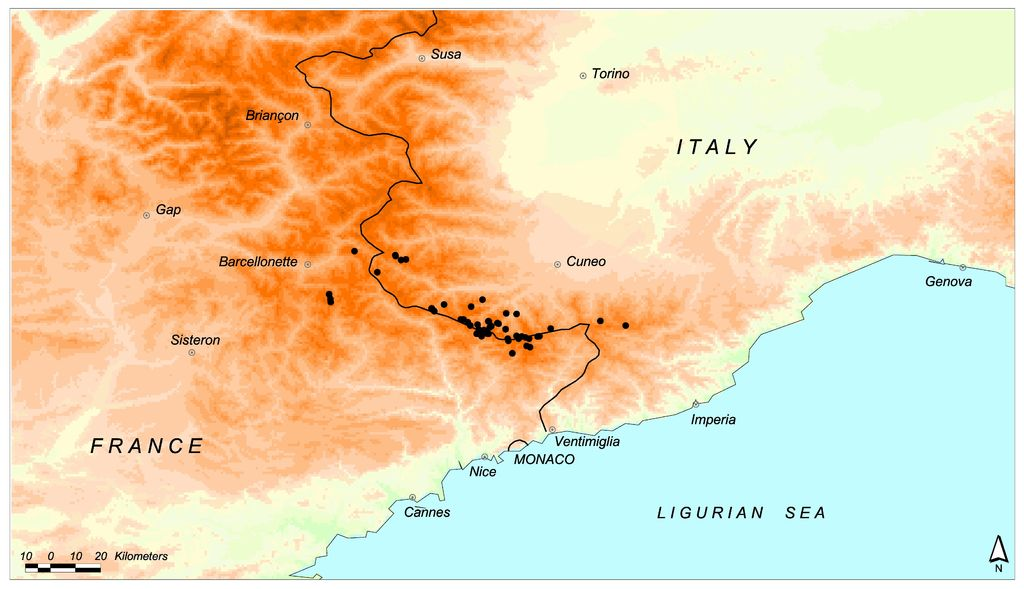
Distribution map of *Vesubia jugorum* (black dots).

Map of records (image): Fig. 3

Map of records (Google Earth): Suppl. material 1

Basis of EOO and AOO: Species Distribution Model

Basis (narrative): We based the Species Distribution Model (SDM) on literature data (Isaia et al. (2015), Isaia et al. (2007), Maurer and Thaler (1988), Simon (1881), Tongiorgi (1968), Tongiorgi (1969)) and original unpublished data gathered during recent surveys (see New occurrences). Occurrences were used to model the current distribution of the species through a MaxEnt model in dismo R package (Hijmans et al. 2014). See Mammola et al. (2015) and Isaia et al. (2016) for details on modeling procedure. We estimated the extent of occurrence (EOO) and the area of occupancy (AOO) from the model, as implemented in the red R package (Cardoso 2016). To estimate the potential variation of the EOO and AOO due to future climate change, the model was projected in the future (year 2028; i.e. 3 spider generations) according to two different representative concentration pathways, namely rcp 2.6 (low emission rate) and rcp 8.5 (high emission rate).

Min Elevation/Depth (m): 2037

Max Elevation/Depth (m): 2939

Range description: Vesubia jugorum was originally described from an unspecified locality at high altitude in the vicinity of St. Martin-Vésubie (Haute Vésubie Valley, France). The range of this stenoendemic species is centered on the Maritime Alps (43 records). Two additional subpopulations occur at the eastern and north-western corners of the range, in Ligurian (2 records) and Cottian Alps (2 records). Most localities are situated in the Site of Community Importance and Special Area of Conservation IT1160056 “Alpi Marittime” (NW Italy).

## New occurrences

### Materials

- **Type status:** Other material. **Occurrence:** recordedBy: Breton, Braud; individualCount: 1; sex: female; lifeStage: adult; **Taxon:** scientificName: Vesubia jugorum; family: Lycosidae; taxonRank: species; scientificNameAuthorship: (Simon, 1881); **Location:** continent: Europe; country: France; stateProvince: Alpes-Maritimes; municipality: Uvernet Fours; locality: Col des Esbéliousses; verbatimElevation: 2500; minimumElevationInMeters: 2500; maximumElevationInMeters: 2500; decimalLatitude: 44.28240; decimalLongitude: 6.71840; georeferenceProtocol: GPS; **Identification:** identifiedBy: Herve; dateIdentified: 2006; **Event:** samplingProtocol: hand collecting; eventDate: 23 Jul 2006; habitat: Rocky areas; **Record Level:** basisOfRecord: PreservedSpecimen
- **Type status:** Other material. **Occurrence:** recordedBy: Breton; individualCount: 1; sex: female; lifeStage: adult; **Taxon:** scientificName: Vesubia jugorum; family: Lycosidae; taxonRank: species; scientificNameAuthorship: (Simon, 1881); **Location:** continent: Europe; country: France; stateProvince: Alpes-Maritimes; municipality: Uvernet Fours; locality: Le Trou de l'Aigle; verbatimElevation: 2748; minimumElevationInMeters: 2748; maximumElevationInMeters: 2748; decimalLatitude: 44.26490; decimalLongitude: 6.72290; georeferenceProtocol: GPS; **Identification:** identifiedBy: Isaia (Validated); dateIdentified: 2016; **Event:** samplingProtocol: hand collecting; eventDate: 20 Aug 2006; habitat: Rocky areas; **Record Level:** basisOfRecord: Based on photographs
- **Type status:** Other material. **Occurrence:** recordedBy: Breton; individualCount: 6; sex: female; lifeStage: adult; **Taxon:** scientificName: Vesubia jugorum; family: Lycosidae; taxonRank: species; scientificNameAuthorship: (Simon, 1881); **Location:** continent: Europe; country: France; stateProvince: Alpes-Maritimes; municipality: Meyronnes; locality: Les Courroies de David; verbatimElevation: 2405; minimumElevationInMeters: 2405; maximumElevationInMeters: 2405; decimalLatitude: 44.43750; decimalLongitude: 6.80880; georeferenceProtocol: GPS; **Identification:** identifiedBy: Isaia (Validated); dateIdentified: 2016; **Event:** samplingProtocol: hand collecting; eventDate: 25 Jun 2007; habitat: Rocky areas; **Record Level:** basisOfRecord: Based on photographs
- **Type status:** Other material. **Occurrence:** recordedBy: Breton; individualCount: 1; sex: male; lifeStage: adult; **Taxon:** scientificName: Vesubia jugorum; family: Lycosidae; taxonRank: species; scientificNameAuthorship: (Simon, 1881); **Location:** continent: Europe; country: France; stateProvince: Alpes-Maritimes; municipality: Allos; locality: Lac de la Petite Cayolle; verbatimElevation: 2600; minimumElevationInMeters: 2600; maximumElevationInMeters: 2600; decimalLatitude: 44.25400; decimalLongitude: 6.72370; georeferenceProtocol: GPS; **Identification:** identifiedBy: Isaia (Validated); dateIdentified: 2016; **Event:** samplingProtocol: hand collecting; eventDate: 03 Aug 2007; habitat: Rocky areas; **Record Level:** basisOfRecord: Based on photographs
- **Type status:** Other material. **Occurrence:** recordedBy: Morando, Pala; individualCount: 5; sex: female; lifeStage: adult; **Taxon:** scientificName: Vesubia jugorum; family: Lycosidae; taxonRank: species; scientificNameAuthorship: (Simon, 1881); **Location:** continent: Europe; country: Italy; stateProvince: Cuneo; municipality: Valdieri; locality: Passo di Préfouns; verbatimElevation: 2395; minimumElevationInMeters: 2395; maximumElevationInMeters: 2395; decimalLatitude: 44.16970; decimalLongitude: 7.22490; georeferenceProtocol: GPS; **Identification:** identifiedBy: Isaia (Validated); dateIdentified: 2016; **Event:** samplingProtocol: vidit; eventDate: 09 Aug 2011; habitat: Rocky areas; **Record Level:** basisOfRecord: Based on photographs
- **Type status:** Other material. **Occurrence:** recordedBy: Giordano, Dalmasso; individualCount: 1; sex: female; lifeStage: adult; **Taxon:** scientificName: Vesubia jugorum; family: Lycosidae; taxonRank: species; scientificNameAuthorship: (Simon, 1881); **Location:** continent: Europe; country: Italy; stateProvince: Cuneo; municipality: Entracque; locality: Sentiero Pagarì; verbatimElevation: 2500; minimumElevationInMeters: 2500; maximumElevationInMeters: 2500; decimalLatitude: 44.13050; decimalLongitude: 7.41020; georeferenceProtocol: GPS; **Identification:** identifiedBy: Isaia (Validated); dateIdentified: 2016; **Event:** samplingProtocol: vidit; eventDate: 17 Aug 2011; habitat: Rocky areas; **Record Level:** basisOfRecord: Based on photographs
- **Type status:** Other material. **Occurrence:** recordedBy: Piacenza; individualCount: 1; sex: female; lifeStage: adult; **Taxon:** scientificName: Vesubia jugorum; family: Lycosidae; taxonRank: species; scientificNameAuthorship: (Simon, 1881); **Location:** continent: Europe; country: France; stateProvince: Alpes-Maritimes; municipality: Saint-Martin-Vesubie; locality: Lake Mercantour; verbatimElevation: 2600; minimumElevationInMeters: 2600; maximumElevationInMeters: 2600; decimalLatitude: 44.14031; decimalLongitude: 7.29012; georeferenceProtocol: GPS; **Identification:** identifiedBy: Isaia (Validated); dateIdentified: 2016; **Event:** samplingProtocol: vidit; eventDate: 02 Aug 2011; habitat: Rocky areas; **Record Level:** basisOfRecord: Based on photographs
- **Type status:** Other material. **Occurrence:** recordedBy: Morando, Pala; individualCount: 1; sex: female; lifeStage: adult; **Taxon:** scientificName: Vesubia jugorum; family: Lycosidae; taxonRank: species; scientificNameAuthorship: (Simon, 1881); **Location:** continent: Europe; country: Italy; stateProvince: Cuneo; municipality: Valdieri; locality: Bivio Lago Nasta; verbatimElevation: 2792; minimumElevationInMeters: 2792; maximumElevationInMeters: 2792; decimalLatitude: 44.16750; decimalLongitude: 7.30020; georeferenceProtocol: GPS; **Identification:** identifiedBy: Isaia (Validated); dateIdentified: 2016; **Event:** samplingProtocol: vidit; eventDate: 30 Aug 2011; habitat: Rocky areas; **Record Level:** basisOfRecord: Based on photographs
- **Type status:** Other material. **Occurrence:** recordedBy: Isaia, Mammola; individualCount: 6; sex: female; lifeStage: adult; **Taxon:** scientificName: Vesubia jugorum; family: Lycosidae; taxonRank: species; scientificNameAuthorship: (Simon, 1881); **Location:** continent: Europe; country: Italy; stateProvince: Cuneo; municipality: Valdieri; locality: Colle di Ciriegia; verbatimElevation: 2543; minimumElevationInMeters: 2543; maximumElevationInMeters: 2543; decimalLatitude: 44.14183; decimalLongitude: 7.28315; georeferenceProtocol: GPS; **Identification:** identifiedBy: Isaia; dateIdentified: 2016; **Event:** samplingProtocol: hand collecting; eventDate: 24 Jun 2016; habitat: Rocky areas; **Record Level:** basisOfRecord: PreservedSpecimen
- **Type status:** Other material. **Occurrence:** recordedBy: Isaia, Mammola; individualCount: 3; sex: female; lifeStage: juvenile, adult; **Taxon:** scientificName: Vesubia jugorum; family: Lycosidae; taxonRank: species; scientificNameAuthorship: (Simon, 1881); **Location:** continent: Europe; country: Italy; stateProvince: Cuneo; municipality: Valdieri; locality: Vallone di Ciriegia; verbatimElevation: 2265; minimumElevationInMeters: 2265; maximumElevationInMeters: 2265; decimalLatitude: 44.14887; decimalLongitude: 7.27741; georeferenceProtocol: GPS; **Identification:** identifiedBy: Isaia; dateIdentified: 2016; **Event:** samplingProtocol: hand collecting; eventDate: 24 Jun 2016; habitat: Rocky areas; **Record Level:** basisOfRecord: PreservedSpecimen
- **Type status:** Other material. **Occurrence:** recordedBy: Isaia, Mammola; individualCount: 1; sex: female; lifeStage: adult; **Taxon:** scientificName: Vesubia jugorum; family: Lycosidae; taxonRank: species; scientificNameAuthorship: (Simon, 1881); **Location:** continent: Europe; country: Italy; stateProvince: Cuneo; municipality: Valdieri; locality: Pian della Casa; verbatimElevation: 2037; minimumElevationInMeters: 2037; maximumElevationInMeters: 2037; decimalLatitude: 44.15387; decimalLongitude: 7.27433; georeferenceProtocol: GPS; **Identification:** identifiedBy: Isaia; dateIdentified: 2016; **Event:** samplingProtocol: hand collecting; eventDate: 24 Jun 2016; habitat: Rocky areas; **Record Level:** basisOfRecord: PreservedSpecimen
- **Type status:** Other material. **Occurrence:** recordedBy: Morando; individualCount: 1; sex: female; lifeStage: adult; **Taxon:** scientificName: Vesubia jugorum; family: Lycosidae; taxonRank: species; scientificNameAuthorship: (Simon, 1881); **Location:** continent: Europe; country: Italy; stateProvince: Cuneo; municipality: Entracque; locality: Bivacco Moncalieri; verbatimElevation: 2710; minimumElevationInMeters: 2710; maximumElevationInMeters: 2710; decimalLatitude: 44.13331; decimalLongitude: 7.39079; georeferenceProtocol: GPS; **Identification:** identifiedBy: Isaia (Validated); dateIdentified: 2016; **Event:** samplingProtocol: vidit; eventDate: 23 Aug 2011; habitat: Rocky areas; **Record Level:** basisOfRecord: Based on photographs
- **Type status:** Other material. **Occurrence:** recordedBy: Mammola; individualCount: 1; sex: female; lifeStage: adult; **Taxon:** scientificName: Vesubia jugorum; family: Lycosidae; taxonRank: species; scientificNameAuthorship: (Simon, 1881); **Location:** continent: Europe; country: Italy; stateProvince: Cuneo; municipality: Vinadio; locality: Alpine scree near Colle della Lombarda; verbatimElevation: 2342; minimumElevationInMeters: 2342; maximumElevationInMeters: 2342; decimalLatitude: 44.21163; decimalLongitude: 7.39079; georeferenceProtocol: GPS; **Identification:** identifiedBy: Mammola (Validated); dateIdentified: 2016; **Event:** samplingProtocol: vidit; eventDate: 20 Jul 2016; habitat: Rocky areas; **Record Level:** basisOfRecord: Based on photographs
- **Type status:** Other material. **Occurrence:** recordedBy: Isaia, Mammola; individualCount: 2; sex: female; lifeStage: adult; **Taxon:** scientificName: Vesubia jugorum; family: Lycosidae; taxonRank: species; scientificNameAuthorship: (Simon, 1881); **Location:** continent: Europe; country: Italy; stateProvince: Cuneo; municipality: Acceglio; locality: Passo dell'Escalon; verbatimElevation: 2939; minimumElevationInMeters: 2939; maximumElevationInMeters: 2939; decimalLatitude: 44.42262; decimalLongitude: 6.95581; georeferenceProtocol: GPS; **Identification:** identifiedBy: Isaia; dateIdentified: 2016; **Event:** samplingProtocol: hand collecting; eventDate: 25 Aug 2016; habitat: Rocky areas; **Record Level:** basisOfRecord: PreservedSpecimen
- **Type status:** Other material. **Occurrence:** recordedBy: Isaia, Mammola; individualCount: 1; sex: female; lifeStage: adult; **Taxon:** scientificName: Vesubia jugorum; family: Lycosidae; taxonRank: species; scientificNameAuthorship: (Simon, 1881); **Location:** continent: Europe; country: Italy; stateProvince: Cuneo; municipality: Canosio; locality: Passo della Gardetta; verbatimElevation: 2568; minimumElevationInMeters: 2568; maximumElevationInMeters: 2568; decimalLatitude: 44.40713; decimalLongitude: 6.99455; georeferenceProtocol: GPS; **Identification:** identifiedBy: Isaia; dateIdentified: 2016; **Event:** samplingProtocol: hand collecting; eventDate: 25 Aug 2016; habitat: Rocky areas; **Record Level:** basisOfRecord: PreservedSpecimen
- **Type status:** Other material. **Occurrence:** recordedBy: Isaia, Mammola; individualCount: 1; sex: female; lifeStage: adult; **Taxon:** scientificName: Vesubia jugorum; family: Lycosidae; taxonRank: species; scientificNameAuthorship: (Simon, 1881); **Location:** continent: Europe; country: Italy; stateProvince: Cuneo; municipality: Acceglio; locality: Oserot; verbatimElevation: 2508; minimumElevationInMeters: 2508; maximumElevationInMeters: 2508; decimalLatitude: 44.40522; decimalLongitude: 6.97708; georeferenceProtocol: GPS; **Identification:** identifiedBy: Isaia; dateIdentified: 2016; **Event:** samplingProtocol: hand collecting; eventDate: 25 Aug 2016; habitat: Rocky areas; **Record Level:** basisOfRecord: PreservedSpecimen
- **Type status:** Other material. **Occurrence:** recordedBy: Isaia, Mammola, Biggi, Galindo; individualCount: 2; sex: female; lifeStage: adult; **Taxon:** scientificName: Vesubia jugorum; family: Lycosidae; taxonRank: species; scientificNameAuthorship: (Simon, 1881); **Location:** continent: Europe; country: Italy; stateProvince: Cuneo; municipality: Argentera; locality: Rocca dei Tre Vescovi; verbatimElevation: 2628; minimumElevationInMeters: 2628; maximumElevationInMeters: 2628; decimalLatitude: 44.36185; decimalLongitude: 6.89117; georeferenceProtocol: GPS; **Identification:** identifiedBy: Isaia; dateIdentified: 2016; **Event:** samplingProtocol: hand collecting; eventDate: 19 Aug 2016; habitat: Rocky areas; **Record Level:** basisOfRecord: PreservedSpecimen
- **Type status:** Other material. **Occurrence:** recordedBy: Milano; individualCount: 5; sex: male, female; lifeStage: adult; **Taxon:** scientificName: Vesubia jugorum; family: Lycosidae; taxonRank: species; scientificNameAuthorship: (Simon, 1881); **Location:** continent: Europe; country: Italy; stateProvince: Cuneo; municipality: Entracque; locality: Vallone del Chiapous; verbatimElevation: 2324; minimumElevationInMeters: 2324; maximumElevationInMeters: 2324; decimalLatitude: 44.17833; decimalLongitude: 7.32277; georeferenceProtocol: GPS; **Identification:** identifiedBy: Isaia; dateIdentified: 2016; **Event:** samplingProtocol: hand collecting; eventDate: 06 Aug 2016; habitat: Rocky areas; **Record Level:** basisOfRecord: PreservedSpecimen
- **Type status:** Other material. **Occurrence:** recordedBy: Isaia, Mammola, Milano; individualCount: 5; sex: male, female; lifeStage: adult; **Taxon:** scientificName: Vesubia jugorum; family: Lycosidae; taxonRank: species; scientificNameAuthorship: (Simon, 1881); **Location:** continent: Europe; country: Italy; stateProvince: Cuneo; municipality: Vinadio; locality: Passo Tesina; verbatimElevation: 2450; minimumElevationInMeters: 2450; maximumElevationInMeters: 2450; decimalLatitude: 44.23121; decimalLongitude: 7.08665; georeferenceProtocol: GPS; **Identification:** identifiedBy: Isaia; dateIdentified: 2016; **Event:** samplingProtocol: hand collecting; eventDate: 20 Jul 2016; habitat: Rocky areas; **Record Level:** basisOfRecord: PreservedSpecimen
- **Type status:** Other material. **Occurrence:** recordedBy: Isaia, Mammola, Milano; individualCount: 5; sex: female; lifeStage: adult; **Taxon:** scientificName: Vesubia jugorum; family: Lycosidae; taxonRank: species; scientificNameAuthorship: (Simon, 1881); **Location:** continent: Europe; country: Italy; stateProvince: Cuneo; municipality: Vinadio; locality: Passo di Sant'Anna; verbatimElevation: 2397; minimumElevationInMeters: 2397; maximumElevationInMeters: 2397; decimalLatitude: 44.22240; decimalLongitude: 7.09513; georeferenceProtocol: GPS; **Identification:** identifiedBy: Isaia; dateIdentified: 2016; **Event:** samplingProtocol: hand collecting; eventDate: 20 Jul 2016; habitat: Rocky areas; **Record Level:** basisOfRecord: PreservedSpecimen
- **Type status:** Other material. **Occurrence:** recordedBy: Milano; individualCount: 4; sex: male, female; lifeStage: adult; **Taxon:** scientificName: Vesubia jugorum; family: Lycosidae; taxonRank: species; scientificNameAuthorship: (Simon, 1881); **Location:** continent: Europe; country: Italy; stateProvince: Cuneo; municipality: Valdieri; locality: Rifugio Bozano; verbatimElevation: 2390; minimumElevationInMeters: 2390; maximumElevationInMeters: 2390; decimalLatitude: 44.18480; decimalLongitude: 7.29145; georeferenceProtocol: GPS; **Identification:** identifiedBy: Isaia; dateIdentified: 2016; **Event:** samplingProtocol: hand collecting; eventDate: 01 Aug 2016; habitat: Rocky areas; **Record Level:** basisOfRecord: PreservedSpecimen
- **Type status:** Other material. **Occurrence:** recordedBy: Isaia, Galindo; individualCount: 4; sex: male, female; lifeStage: adult; **Taxon:** scientificName: Vesubia jugorum; family: Lycosidae; taxonRank: species; scientificNameAuthorship: (Simon, 1881); **Location:** continent: Europe; country: Italy; stateProvince: Cuneo; municipality: Chiusa Pesio; locality: Canale dei Torinesi (Monte Marguareis); verbatimElevation: 2160; minimumElevationInMeters: 2160; maximumElevationInMeters: 2160; decimalLatitude: 44.17671; decimalLongitude: 7.69461; georeferenceProtocol: GPS; **Identification:** identifiedBy: Isaia; dateIdentified: 2016; **Event:** samplingProtocol: hand collecting; eventDate: 21 Aug 2016; **Record Level:** basisOfRecord: PreservedSpecimen

## Extent of occurrence

EOO (km2): 4412

Trend: Decline (projected)

Justification for trend: The species inhabits rocky areas of the subnival and nival zones of the Maritime Alps. The high altitude regions are particularly vulnerable to climatic variations due to climate change, with warming rates approximately doubling the global average (Böhm et al. 2001). In consequence of temperature increase, range shifts towards higher latitudes or altitudes are expected (Bellard et al. 2012), causing a decrease in the EOO. In particular, we predict a future reduction of the EOO ranging from 24% (low emission scenario) to 41% (high emission scenario).

Future decline (%): 41

Causes ceased?: No

Causes understood?: Yes

Causes reversible?: No

Extreme fluctuations?: Unknown

## Area of occupancy

Trend: Decline (projected)

Justification for trend: Future forecasts based on different emission scenarios show a significant reduction in the bioclimatic range of Vesubia jugorum (details in Isaia et al. (2016)). We predict a future reduction of the AOO ranging from 12% (low emission scenario) to 32% (high emission scenario). In this perspective, it is important to take into account the connectivity of the suitable habitat, in order to provide coherent interpretations of the future trends. Suitable ecological corridors for this species would be represented by high-altitude rocky areas, which rarely occur between the Maritime Alps and the northern Alpine districts. The species is probably also limited by its low dispersal ability.

Future decline (%): 32

Causes ceased?: No

Causes understood?: Yes

Causes reversible?: No

Extreme fluctuations?: Unknown

AOO (km2): 835

## Locations

Number of locations: 1﻿﻿﻿

Justification for number of locations: The whole population is affected by the ongoing climate change.

Trend: Stable

Extreme fluctuations?: Unknown

## Population

Number of individuals: Unknow﻿n —a census/estimation﻿ of the population has never been attempted.﻿

Trend: Decline (inferred)

Justification for trend: Inferred from the decline in AOO.

Causes ceased?: No

Causes understood?: Yes

Causes reversible?: No

Extreme fluctuations?: Unknown

## Subpopulations

Number of subpopulations: 3

Trend: Decline (projected)

Justification for trend: The main subpopulation of the species is centred in Maritime Alps. This core area includes over 90% of the known localities. Two additional subpopulations are identified at the north-western and eastern corners of the distribution range, corresponding to the Southern Cottian and Ligurian Alps, respectively. All future warming scenarios predict the extinction of the latter subpopulation.

Extreme fluctuations?: Unknown

Severe fragmentation?: No

Justification for fragmentation: Within the core area the suitable habitat is roughly continuous, with peaks and rocky areas ensuring connectivity between local populations. However, unsuitable habitat —namely grasslands at lower altitudes— reduces connectivity between subpopulations.

## Habitat

System: Terrestrial

Habitat specialist: Yes

**Figure 4.**
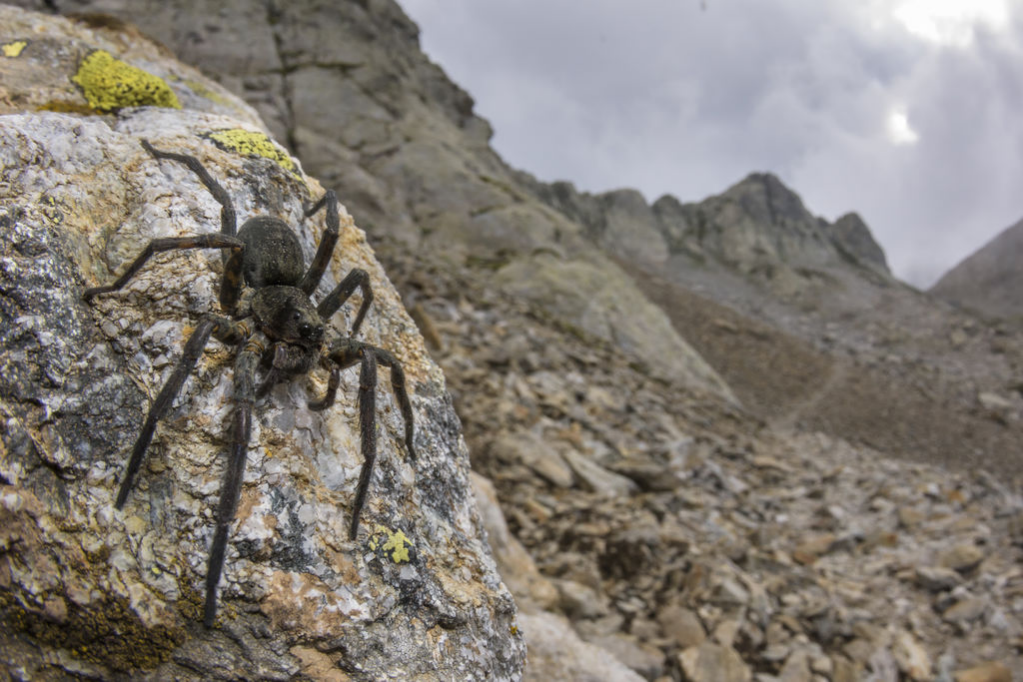
Typical rocky areas inhabited by *Vesubia jugorum* — Maritime Alps, Province of Cuneo. [Photo credit: Emanuele Biggi, 2016]

**Figure 5.**
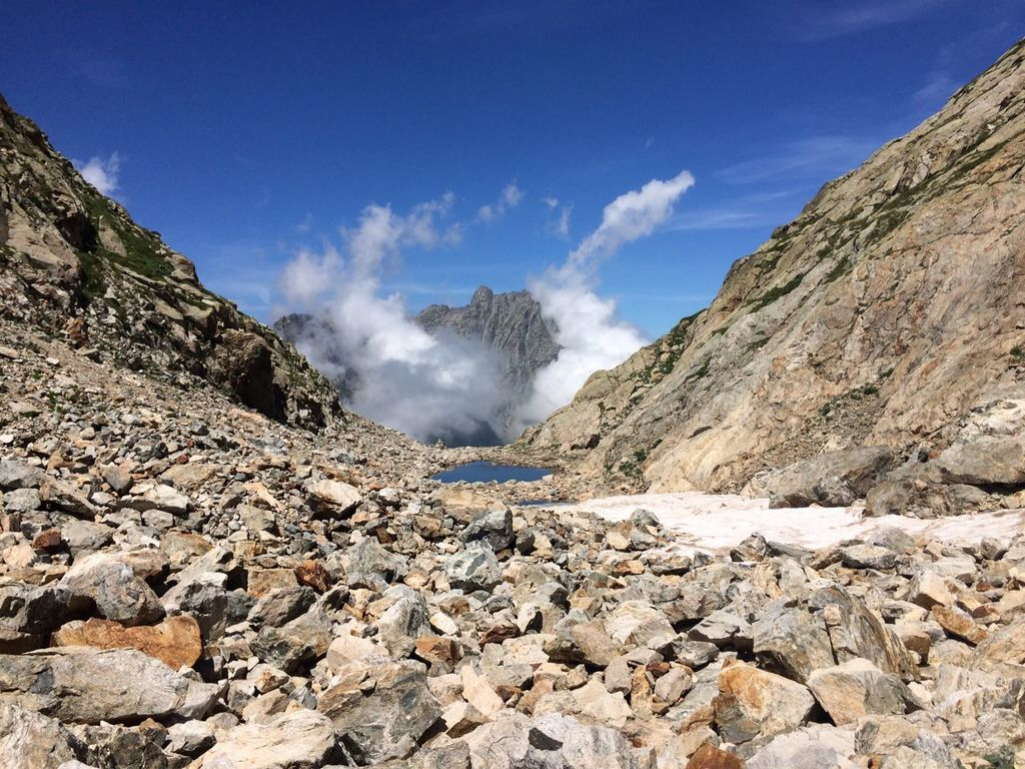
An example of rocky areas colonized by *Vesubia jugorum* — Maritime Alps, Province of Cuneo. [Photo credit: Filippo Milano, 2016]

Habitat (narrative): The species is restricted to alpine rocky areas above 2,000 m. These include rocky debris, boulder fields, and alpine screes (Figs 4, 5).

Trend in extent, area or quality?: Stable

Justification for trend: The optimal habitat for the species is not expected to undergo significant variations in the future, as touristic pressure is negligible in the high mountain peaks of the Maritime Alps. Altitudinal shifts of vegetation due to climate change may hypothetically affect the extension of the subnival zone determining small variations in the extent of high altitude rocky areas.

Figure(s) or Photo(s): Fig. 5

### Habitat

Habitat importance: Major Importance

Habitats: 6. Rocky areas (e.g. inland cliffs, mountain peaks)

## Ecology

Size: Bod﻿y length: 15﻿–﻿20 mm, ﻿prosoma﻿: 7–9 mm

Generation length (yr): 4

Dependency of single sp?: No

Ecology and traits (narrative): The species is active during the warm season, from the snow melting in late June to mid September. Males are mainly found from July to August, whereas females and immatures can be found throughout the whole season. During the day, individuals can be observed wandering on the rocks. Females with cocoons build retreats under stones (see illustration in Tongiorgi (1969)), usually of 10–15 x 3cm. Preliminary data on reared individuals and estimations based on body size suggest generation length > 3–4 years.

## Threats

### Threats

Threat type: Future

Threats: 11. Climate change & severe weather

## Conservation

Justification for conservation actions: Most of the species range falls within the borders of national parks, sites of community importance and special protection areas, namely Parco Naturale Alpi Marittime (Italy), Parco Naturale del Marguareis (Italy) and Parc National du Mercantour (France).In the light of the existing threats, it is expected that the survival of the species will depend on monitoring, conservation management and translocation programmes. Ex-situ conservation should also be considered, to ensure the preservation of healthy individuals for re-introduction in suitable habitats situated to the north from the current distribution area.

### Conservation actions

Conservation action type: In Place

Conservation actions: 1.1. Land/water protection - Site/area protection

### Conservation actions

Conservation action type: Needed

Conservation actions: 3. Species management

## Other

Justification for ecosystem services : 

### Research needed

Research needed: 1. Research

Justification for research needed: Little is known about the ecological requirements of the species, life history, phenology and potential interactions with other ecosystem components. Population size need to be estimated as well as dispersal ability.

## Supplementary Material

Supplementary material 1 Extent of Occurrence of ﻿﻿*Vesubia﻿ jugorum*﻿﻿ (Simon, 1881) Data type: Geographic range
File: oo_103034.kml Stefano Mammola, Filippo Milano﻿, Pedro Cardoso, Marco Isaia
